# Clinicopathological features and CCT2 and PDIA2 expression in gallbladder squamous/adenosquamous carcinoma and gallbladder adenocarcinoma

**DOI:** 10.1186/1477-7819-11-143

**Published:** 2013-06-19

**Authors:** Qiong Zou, Zhu-lin Yang, Yuan Yuan, Jing-he Li, Lu-feng Liang, Gui-xiang Zeng, Sen-lin Chen

**Affiliations:** 1 Department of Pathology, Third Xiangya Hospital, Central South University, Changsha, Hunan 410013, PR China; 2 Research Laboratory of Hepatobiliary Diseases, Second Xiangya Hospital, Central South University, Changsha, Hunan 410011, PR China; 3 Department of Pathology, Basic School of Medicine, Central South University, Changsha, Hunan 410078, PR China; 4 Department of Hepatobiliary and Pancreatic Surgery, Hunan Provincial People’s Hospital, Changsha, Hunan 410007, PR China; 5 Department of Pathology, Loudi Central Hospital, Loudi, Hunan 417011, PR China; 6 Department of Pathology, Hunan Provincial Tumor Hospital, Changsha, Hunan 410013, PR China

**Keywords:** gallbladder cancer, adenocarcinoma, squamous cell carcinoma, adenosquamous carcinoma, CCT2, PDIA3

## Abstract

**Background:**

Gallbladder cancer (GBC) is a relatively uncommon carcinoma among gastrointestinal cancers and usually has a rather poor prognosis. The most common subtype of GBC is adenocarcinoma (AC), which accounts for about 90% of GBC. Squamous carcinoma/adenosquamous carcinoma (SC/ASC) are comparatively rare histopathological subtypes of GBC. The clinicopathological features and biological behaviors of SC/ASC have not been well-characterized. No molecular biomarkers are currently available for predicting the progression, metastasis, and prognosis of the SC/ASC subtype of GBC.

**Methods:**

We examined the expression levels of CCT2 and PDIA3 by immunohistochemistry (IHC) staining in human GBC tissue samples collected from 46 patients with SC/ASC and evaluated the clinicopathological significance of both CCT2 and PDIA3 expression in the SC/ASC subtypes of GBC by Kaplan-Meier analysis and multivariate Cox regression analysis. For comparison, we included specimens from 80 AC patients in our study to investigate the specificity of CCT2 and PDIA3 expression in GBC subtypes.

**Results:**

We found that the positive expression of CCT2 and PDIA3 was significantly associated with clinicopathological features of both SC/ASC and AC specimens, including high TNM stage and lymph node metastasis. Univariate analysis revealed that the two-year survival rate was significantly lower for patients with positive expression of CCT2 and PDIA3 than for those with negative expression. Multivariate analysis also indicated that the positive expression of CCT2 and PDIA3 was negatively correlated with poor postoperative patient survival and positively correlated with high mortality.

**Conclusions:**

Our study suggests that positive expression of CCT2 or PDIA3 is associated with tumor progression and the clinical behavior of gallbladder carcinoma. Therefore, CCT2 and PDIA3 could be potentially important diagnostic and prognostic biomarkers for both SC/ASC and AC subtypes of GBC.

## Background

Gallbladder cancer (GBC) is a relatively uncommon gastrointestinal carcinoma, but its incidence is increasing worldwide and its prognosis is rather poor [1]. Early diagnosis and radical resection, when possible, are helpful in improving prognosis. However, it is usually impossible to diagnose GBC at its early stages due to the lack of obvious and specific symptoms. Common symptoms do not develop until GBC is at an advanced stage and include steady pain and discomfort in the upper right abdomen. Clinically, over 90% of GBC patients are diagnosed at an advanced stage when the outcome of surgical therapy is very poor. Most patients die within one year after diagnosis [2]. The majority of GBC is adenocarcinoma (AC), accounting for about 90% of GBC, while the squamous cell carcinoma/adenosquamous carcinoma (SC/ASC) are very uncommon and only account for 1.4% to 10.4% of GBC [1]. Because of the low incidence of SC/ASC, their clinicopathological features and biological behaviors have not been well characterized. Most previous reports focus on patient summaries or survival analyses [3-8]. To date, no molecular biomarkers are available for SC/ASC to predict their progression, metastasis, and prognosis.

CCT2 (chaperonin containing TCP1, subunit 2) is a molecular chaperone, a subunit of the TCP1 complex, which helps the correct folding of many proteins such as actin and tubulin which are important in maintaining the dynamic equilibrium of cell stability [9-15]. The overexpression of CCT2 is highly related to the tumorigenesis, tumor progression, and prognosis of some cancers, including hepatocellular carcinoma and colonic carcinoma [16-18]. In addition, the expression of CCT2 is significantly higher in gastric carcinoma than in normal gastric mucosa [19]. Increased expression of CCT2 also contributes to tumor cell resistance to chemotherapy and radiation therapy [20].

PDIA3 (protein disulfide isomerase A3) is a member of the disulfide isomerase family proteins [21,22] and is a component of the molecular chaperone complex containing calnexin and calreticulin, which promotes the correct folding of newly synthesized glycoproteins in the endoplasmic reticulum [21,22]. In the endoplasmic reticulum, PDIA3 facilitates the formation of disulfide bonds in glycoproteins through its redox isomerase activity. The expression level of PDIA3 in cancer cells is linked to tumor progression and prognosis of some human tumors. For example, PDIA3 expression is increased in ovarian cancer cells and has been considered as a potential biomarker for ovarian cancer prognosis [23]. PDIA3 expression in hepatocellular carcinoma is positively correlated with tumor grade and alpha-fetoprotein (AFP) level [24]. Down-regulation of PDIA3 by siRNA can inhibit the proliferation of breast cancer cells [25]. In addition, PDIA3 expression in cancer cells is critical for tumor cell resistance to thermotherapy in A549 and UO31 cancer cell lines [26]. Therefore, the high level of PDIA3 expression is an important potential biomarker for rapid tumor progression and poor prognosis.

Although their clinical importance was well studied in other cancers, the expression of CCT2 and PDIA3 and their clinicopathological significance in gallbladder cancer have never been investigated. In this study, we determined the expression levels of CCT2 and PDIA3 by immunohistochemistry staining in tumor samples from 46 SC/ASC and 80 AC patients, and then investigated the relationship between these expression levels and tumor progression as well as the prognosis.

## Methods

### Patients

We collected data and tumor specimens from 46 patients diagnosed with gallbladder SC/ASC (26 SC patients and 20 ASC patients) who were surgically treated in seven local hospitals between January 1995 and December 2009. These patients accounted for 4.34% (46/1,060) of all cases of gallbladder cancer registered in the same hospitals during the same period. For comparison, we also collected tissue samples from 80 patients with gallbladder AC from these hospitals during the period from January 2000 to December 2009. The study was approved by the Ethics Committee of Human Study of Central South University.

Among the 80 AC patients, 26 patients were male and 54 patients were female, with a median age of 53.8 ± 9.9 (from 33 to 80) years. The pathological type (carcinoma differentiation) included 27 cases with well-differentiated carcinoma, 25 with moderately-differentiated carcinoma, and 28 with poorly-differentiated carcinoma. Fifty (62.5%) patients had tumors ≤ 3 cm in diameter, and 30 (37.5%) had tumors > 3 cm. Gallstones were identified as a companion factor in 38 patients (47.5%). Forty nine patients (61.3%) had their tumor with adjacent tissue invasion, and 50 patients (62.5%) had their tumor with regional lymph node metastasis. Staging analysis revealed that 8 cases were TNM stage I, 13 were stage II, 38 were stage III, and 21 were stage IV. 26 patients (32.5%) underwent radical resections, 28 patients (35.0%) underwent palliative resections, and 26 patients (32.5%) underwent biopsies without complete tumor removal.

Among the 80 AC patients, 26 patients were male and 54 patients were female, with a median age of 53.8±9.9 (from 33 to 80) years. The pathological type (carcinoma differentiation) included 27 cases with well-differentiated carcinoma, 25 with moderately-differentiated carcinoma, and 28 with poorly-differentiated carcinoma. Fifty (62.5%) patients had tumors ≤3 cm in diameter, and 30 (37.5%) had tumors >3 cm. Gallstones were identified as a companion factor in 38 patients (47.5%). Forty nine patients (61.3%) had their tumor with adjacent tissue invasion, and 50 patients (62.5%) had their tumor with regional lymph node metastasis. Staging analysis revealed that 8 cases were TNM stage I, 13 were stage II, 38 were stage III, and 21 were stage IV. 26 patients (32.5%) underwent radical resections, 28 patients (35.0%) underwent palliative resections, and 26 patients (32.5%) underwent biopsies without complete tumor removal.

The 46 patients with SC/ASC and 80 patients with AC were followed for two years after surgery through mail and phone calls. Among the 46 SC/ASC patients, 33 patients survived for < one year after surgery and 13 patients survived for ≥ one year, of whom only 4 survived for more than two years. Average survival time for the SC/ASC patients was 10.07 ± 0.78 months. Of the 80 AC patients, 57 patients survived for < one year after surgery and 23 patients survived for ≥ one year, of whom only 9 survived for more than two years. Average survival time for AC patients was 10.34 ± 0.63 months.

### Specimen processing and immunohistochemistry (IHC) staining

Surgically obtained specimens were fixed with 4% formaldehyde for 24 hours and then embedded in paraffin. The paraffin-embedded tissues were sectioned at 4 μm thickness for immunostaining with CCT2 and PDIA3 antibodies using peroxidase-based EnVision™ Detection kit (Dako Laboratories, Carpinteria, CA, USA). Rabbit anti-CCT2 and rabbit anti-PDIA3 antibodies were purchased from Abgent Company (San Diego,, USA). The percentage of positive cells (brown cytoplasmic and/or nuclear staining) was calculated from 400 cells in 10 randomly selected fields. Cases with ≥ 25% positive cells were considered positive while cases with < 25% positive cells were considered negative [27-29]. For a positive control, a biopsy with positive expression of CCT2 and PDIA3 from Beijing Zhongshan Biotechnology Company (Beijing, China) was used, and for a negative control the primary antibody was replaced by 5% FBS for the staining.

### Statistical methods

Data were analyzed using the SPSS13.0 statistical package from SPSS Inc (Chicago, USA).. The correlation between the expression of CCT2 or PDIA3 with the histological or clinical factors was analyzed using Chi square (χ^2^) or Fisher’s exact test. Univariate survival analysis was performed using the Kaplan-Meier method and log-rank test. Cox proportional hazards regression model and Wald’s test were used for multivariate survival analysis and for determining the 95% confidence intervals. *P* values < 0.05 were considered to be statistically significant.

## Results

### Clinicopathological features of SC/ASC and AC

As shown in Table 1, GBC was diagnosed more frequently in older patients (> 45 years) than in those young patients (≤ 45 years), and this was true for both SC/ASC (93.5%) and AC (80%). However, tumors with poor differentiation were more frequently identified in AC patients than in SC/ASC patients (*P* < 0.05) (Table 1). On the other hand, the percentage of patients with tumor mass > 3 cm was higher in SC/ACS patients (56.5%) than in AC patients (37.5) (*P* < 0.05). There were no significant differences in gender, presence of gallstones, TNM stage, lymph node metastases, adjacent tissue invasion, surgical procedure, or average survival time between patients with SC/ASC and AC (*P* > 0.05).

**Table 1:** The CCT2 and PDIA3 expression and clinicopathological features of gallbladder SC/ASC and AC

**Clinicalpathological features**	**SC/ASC (n = 46)**	**AC (n = 80)**	**χ**^**2**^	***P***
Gender				
male	19(41.3)	26(32.5)	0.986	0.352
female	27(58.7)	54(67.5)		
Age (years)				
≤ 45	3(6.5)	16(20.0)	4.143	0.042
> 45	43(93.5)	64(80.0)		
Degree of differentiation				
well-differentiated	16(34.8)	27(33.8)	8.515	0.014
moderately-Differentiated	24(52.2)	25(31.3)		
poorly-differentiated	6(13.0)	28(35.0)		
Tumor maximum diameter (cm)				
≤ 3 cm	20(43.5)	50(62.5)	4.280	0.039
> 3 cm	26(56.5)	30(37.5)		
Gallbladder stones				
no	18(39.1)	42(52.5)	2.093	0.148
yes	28(60.9)	38(47.5)		
TNM stage				
I+II	12(26.1)	21(26.3)	0.287	0.866
III	20(33.5)	38(47.5)		
IV	14(30.4)	21(26.3)		
Lymph node metastasis				
no	17(37.0)	30(37.5)	0.004	0.952
yes	29(63.0)	50(62.5)		
Invasion				
no	16(34.8)	31(38.8)	0.197	0.658
yes	30(62.5)	49(61.3)		
Surgical				
radical	14(30.4)	26(32.5)	0.215	0.898
palliative	18(39.1)	28(35.0)		
biopsy	14(30.4)	26(32.5)		
average survival time	10.07(4 to 25)	10.34(3 to 27)	0.014	0.906
CCT2				
-	23(50.0)	31(46.2)	0.165	0.685
+	23(50.0)	49(53.8)		
PDIA3				
-	20(43.5)	35(43.7)	0.001	0.976
+	26(56.5)	45(56.3)		

### Expression of CCT2 and PDIA3 in SC/ASC and AC

**Figure 1:**
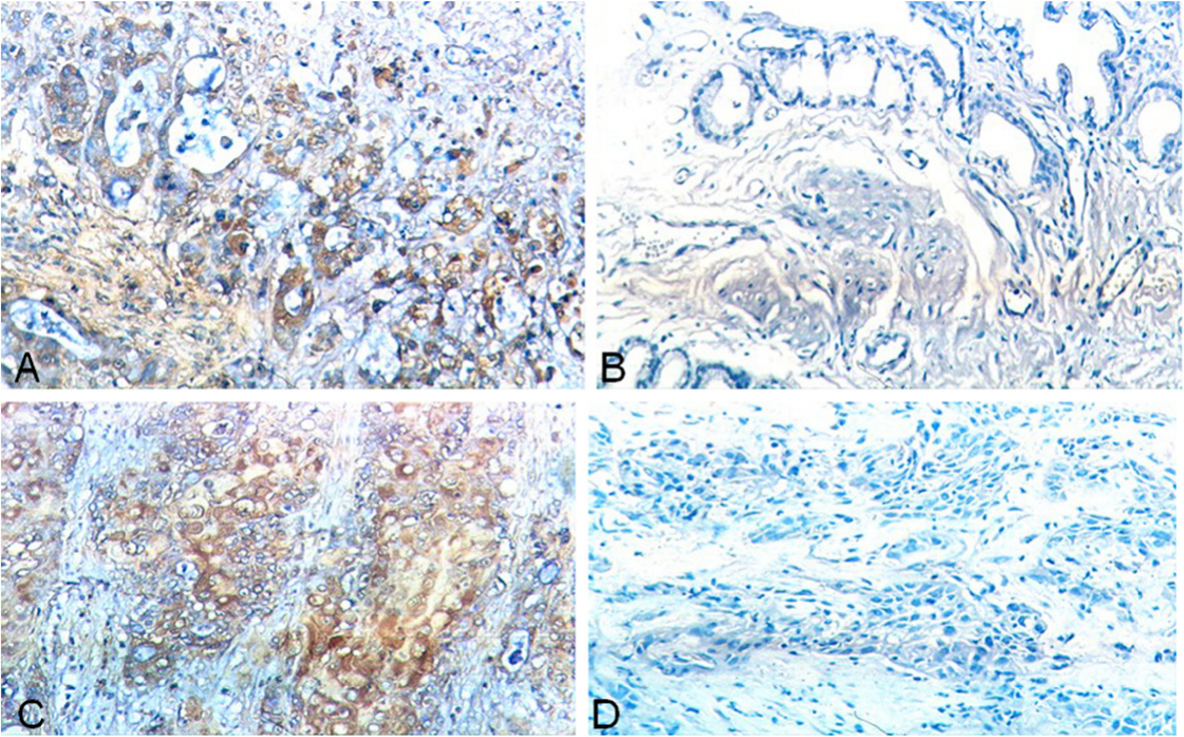
**Expression of CCT2 and PDIA3 in gallbladder SC/ASC by IHC staining using EnVision system. (A)** CCT2 positive expression in poorly-differentiated gallbladder ASC. **(B)** CCT2 negative expression in well-differentiated gallbladder ASC. **(C)** PDIA3 positive expression in poorly-differentiated gallbladder SC. **(D)** PDIA3 negative expression in moderately-differentiated gallbladder SC. Original magnification ×200.

**Figure 2:**
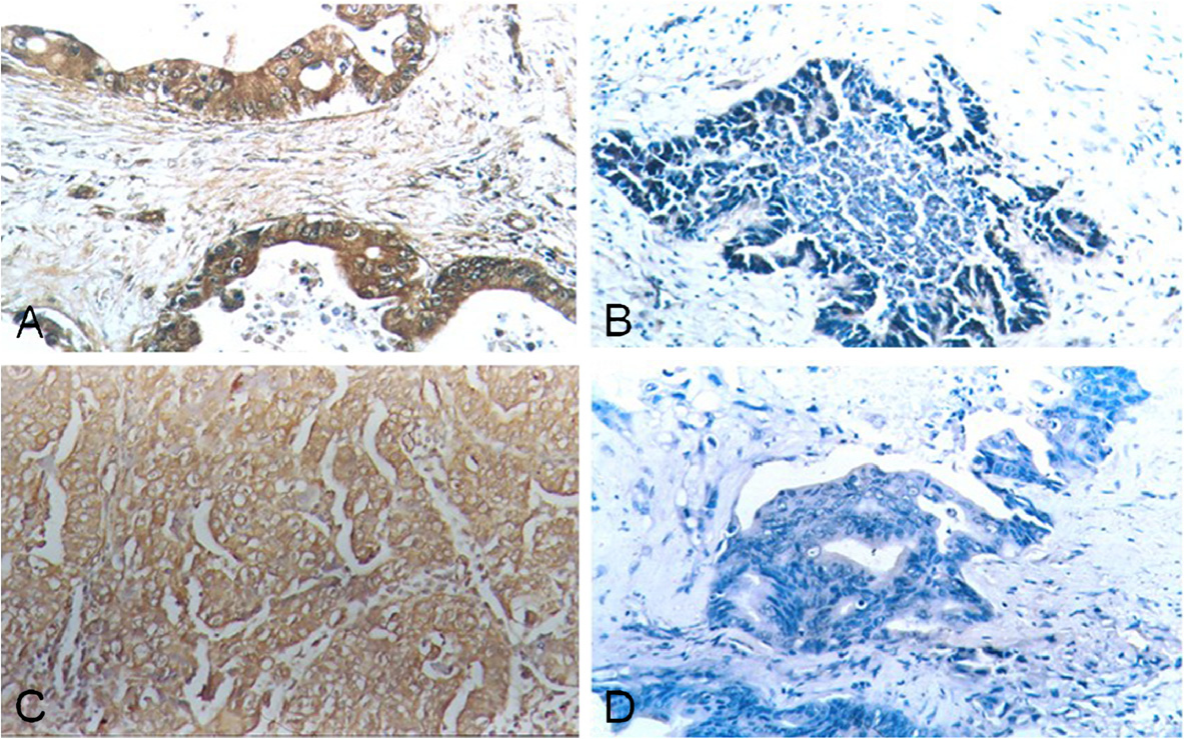
**Expression of CCT2 and PDIA3 in gallbladder AC by IHC using EnVision system. (A)** CCT2 positive expression in poorly-differentiated gallbladder AC. **(B)** CCT2 negative expression in well-differentiated gallbladder AC. **(C)** PDIA3 positive expression in poorly-differentiated gallbladder AC. **(D)** PDIA3 negative expression in well-differentiated gallbladder AC. Original magnification ×200.

The expression of CCT2 and PDIA3 by immunohistochemical (IHC) staining was detected predominately in the cytoplasm and occasionally in the nucleus (Figures 1 and 2). Among 46 SC/ASC tissue samples, 23 (50%) samples showed CCT2 positive expression and 26 (56.5%) samples had PDIA3 positive expression (Table 1). In the 80 AC specimens, we found that 43 (53.8%) samples expressed CCT2 and 45 (56.3%) expressed PDIA3 (Table 1). There were no differences in the expression patterns of CCT2 or PDIA3 between the different GBC subtypes (*P* > 0.05).

### Relationship between CCT2 and PDIA3 expression and clinicopathological features of SC/ASC and AC subtypes

As shown in Table 2, the expression of CCT2 in tissue samples of SC/ASC significantly correlated with tumor size, TNM stage, and lymph node metastasis status. The percentage of tumor samples with CCT2 expression detected by IHC was significantly different between large tumors and small tumors. 65.4% of large tumors (tumor > 3 cm) had CCT2 expression whereas only 30% of small tumors (tumor ≤ 3 cm) showed CCT2 expression. Moreover, 85.7% of stage IV tumor samples expressed CCT2, which was significantly higher than that of stage III (40%) and stage I+II (20%) tumor samples. In addition, CCT2 expression was detected more frequently in tumors with lymph node metastasis (62.1%) than those without lymph node metastasis (29.4%) (Table 2, *P* = 0.03). There was no significant correlation between CCT2 expression and patient gender, age, histopathological types, gallstones, adjacent tissue invasion, or surgical procedure (*P* > 0.05).

**Table 2:** The relationship between CCT2 and PDIA3 expression and clinicopathological features of gallbladder SC/ASC

**Clinicalpathological features**	**Case number**	**CCT2**			**PDIA3**		
		**Positive number (%)**	**χ**^**2**^	***P***	**Positive number (%)**	**χ**^**2**^	***P***
Gender							
male	19	7(36.8)	2.242	0.134	12(63.2)	0.580	0.446
female	27	16(59.3)			14(51.9)		
Age (years)							
≤ 45	3	1(33.3)	0.357	0.550	1(33.3)	0.702	0.402
> 45	43	22(51.2)			25(58.1)		
Pathological types							
SC	26	14(53.8)	0.354	0.552	18(69.2)	3.930	0.047
ASC	20	9(45.0)			8(40.0)		
Degree of differentiation^a^							
well-differentiated	16	8(50.0)	3.333	0.189	8(50.0)	5.940	0.051
moderately-differentiated	24	10(41.7)			12(50.0)		
poorly-differentiated	6	5(83.3)			6(100.0)		
Tumor maximum diameter (cm)							
≤ 3 cm	20	6(30.0)	5.121	0.020	9(45.0)	1.911	0.167
> 3 cm	26	17(65.4)			17(65.4)		
Gallbladder stones							
no	18	10(55.6)	0.365	0.546	12(66.7)	0.351	0.554
yes	28	13(46.4)			14(50.0)		
TNM stage							
I + II	12	3(25.0)			5(41.7)		
III	20	8(40.0)	10.943	0.005	9(45.0)	7.013	0.028
IV	14	12(85.7)			12(85.7)		
Lymph node metastasis							
no	17	5(29.4)	4.572	0.033	6(35.5)	4.945	0.026
yes	29	18(62.1)			20(69.0)		
Invasion							
no	16	5(31.3)	3.450	0.063	5(31.3)	6.376	0.012
yes	30	18(60.0)			21(70.0)		
Surgical							
radical	14	4(28.6)	5.143	0.076	6(42.8)	6.987	0.030
palliative	18	9(50.0)			8(44.4)		
biopsy	14	10(71.4)			12(85.7)		

^a^Comparison between well-differentiated and moderately-differentiated SC/ ASC: *P*_CCT2_ = 0.333; *P*_PDIA3_ = 0.051; Comparison between moderately-differentiated and poorly-differentiated SC/ ASC: *P*_CCT2_ = 0.169; *P*_PDIA3_ = 0.057.

In contrast to CCT2 expression, which showed no significant differences between AC and ASC, PDIA3 was expressed more frequently in SC than ASC (Table 2, *P* = 0.047). Moreover, a higher expression of PDIA3 significantly correlated with higher TNM stage, adjacent tissue invasion, and lymph node metastasis (Table 2, *P* < 0.05).

Similar findings were observed in the AC subtype of GBC. As shown in Table 3, the poorer the differentiation of tumors, the higher the percentage of tumors with CCT2 expression. In addition, the higher the TNM stage of AC, the higher the percentage of tumors with CCT2 expression (Table 3, *P* < 0.05). Furthermore, we observed a significant correlation between the expression of CCT2 and tumor size, invasion status, and lymph node metastasis of AC as well as patient age (Table 3, *P* < 0.05).

**Table 3:** The relationship between CCT2 and PDIA3 expression and clinicopathological features of gallbladder AC

**Clinical and pathological features**	**Case number**	**CCT2**			**PDIA3**		
		**Positive number (%)**	**χ**^**2**^	***P***	**Positive number (%)**	**χ**^**2**^	***P***
Gender							
male	26	14(53.8)	0.000	0.990	14(53.8)	0.090	0.764
female	54	29(53.7)			31(57.4)		
≤ 45							
> 45	16	5(31.3)	4.073	0.044	7(43.8)	1.270	0.260
Degree of differentiation^a^	64	38(59.4)			38(59.4)		
well-differentiated							
moderately-Differentiated	27	10(37.0)	5.326	0.070	12 (44.4)	11.840	0.000
poorly-differentiated	25	14(56.0)			10(40.0)		
Tumor maximum diameter (cm)	28	19(67.9)			23(82.1)		
≤ 3 cm							
> 3 cm	50	22(44.0)	5.099	0.024	23(46.0)	5.692	0.017
Gallbladder stones	30	21(70.0)			22(73.3)		
no							
yes	42	19(45.2)	2.577	0.108	25(59.5)	0.343	0.558
TNM stage	38	24(63.2)			20(52.6)		
I + II							
III	21	6(28.6)			7(33.3)		
IV	38	20(52.6)	11.625	0.003	20(52.6)	12.092	0.002
Lymph node metastasis	21	17(81.0)			18(85.7)		
no							
yes	30	10(33.3)	8.049	0.004	12(40.0)	5.150	0.020
Invasion	50	33(66.0)			33(66.0)		
no							
yes	31	11(35.5)	6.793	0.009	14(45.2)	2.529	0.112
Surgical	49	32(65.3)			31(63.3)		
radical							
palliative	26	8(30.8)	11.140	0.004	10(38.5)	7.940	0.019
biopsy	28	15(53.6)			15(53.6)		
≤ 45	26	20(76.9)			20(76.9)		

^a^Comparison between well-differentiated and poorly-differentiated adenocarcinoma: χ^2^_CCT2_ = 5.24, *P* = 0.022; χ^2^_PDIA3_ = 8.44, *P* = 0.004; Comparison between moderately-differentiated and poorly-differentiated adenocarcinoma: χ^2^_CCT2_ = 0.79, *P* = 0.374; χ^2^_PDIA3_ = 9.98, *P* = 0.002.

In contrast to the expression of PDIA3 in SC/ASC, we found that the expression of PDIA3 in AC significantly correlated not only with large tumor size, higher TNM stage, and lymph node metastasis, but also with poor differentiation (Table 3, *P* < 0.05). However, no correlation between PDIA3 expression and adjacent tissue invasion was observed in AC analyzed (Table 3, *P* > 0.05).

### Impact of CCT2 and PDIA3 expression on overall survival of SC/ASC and AC patients

To further understand the importance of CCT2 and PDIA3 expression for SC/ASC and AC of GBC, we analyzed the survival data of all 46 SC/ASC and 80 AC patients after two years of following-up. Patients surviving longer than two years were censored from the statistical analysis. We chose two years as the mark point for our survival data analysis because a majority of the patients died within two years of surgery.

**Figure 3:**
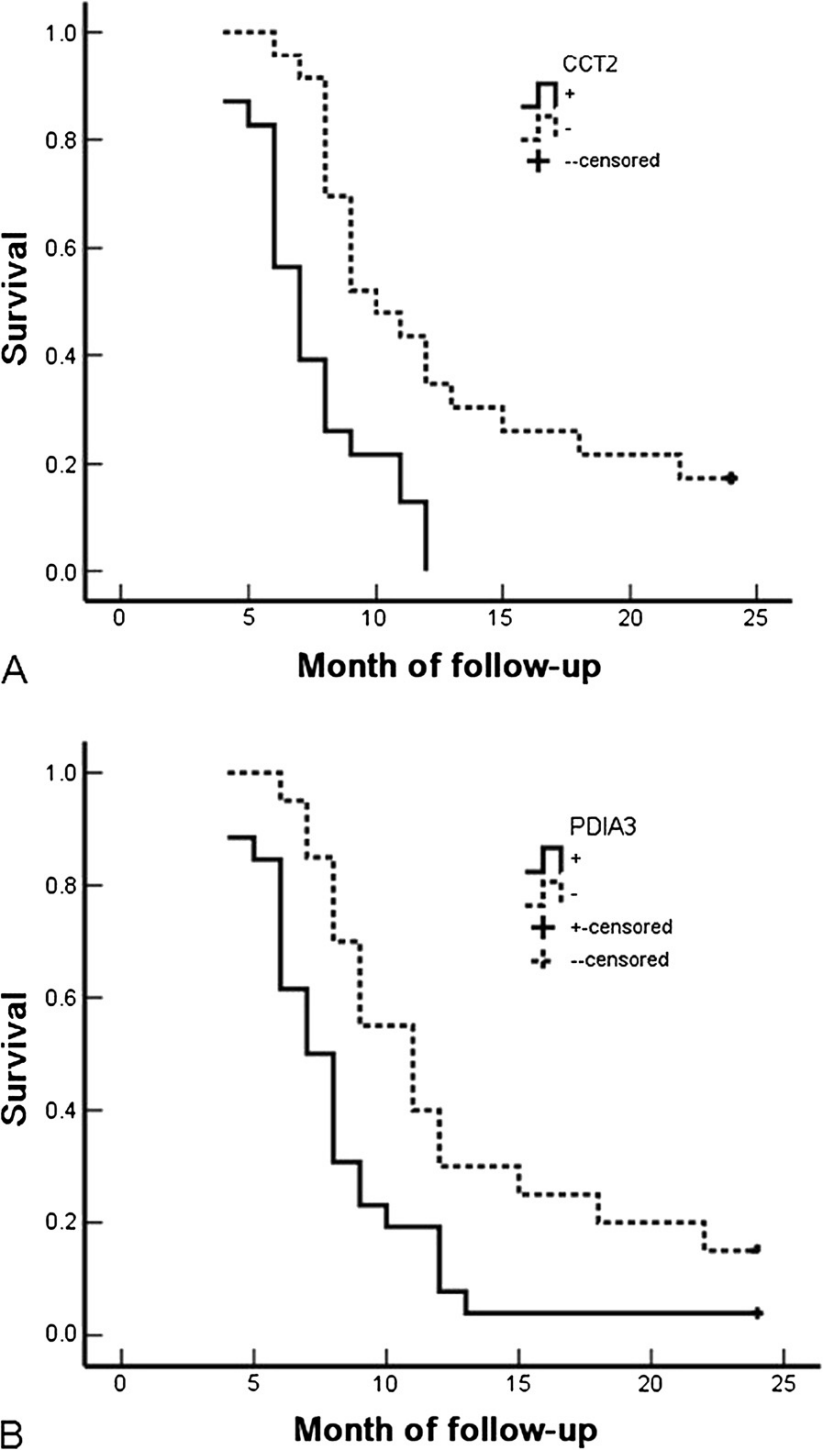
**Correlation between positive expression of CCT2 or PDIA3 and overall survival of patients with gallbladder SC/ASC. A**: Kaplan-Meier analysis of overall survival of gallbladder SC/ASC patients according to the CCT2 expression in their cancers (p < 0.001). **B**: Kaplan-Meier analysis of overall survival of gallbladder SC/ASC patients according to PDIA3 expression in their cancers (p = 0.008).

We found a significant correlation between CCT2 and PDIA3 expression and the survival rate of SC/ASC patients. The survival time of patients with CCT2 and PDIA3 expression in their tumors was significantly shorter than that of patients without CCT2 and PDIA3 expression in their tumors (*P* < 0.001 and *P* = 0.008 for CCT2 and PDIA3, respectively) (Table 4 and Figure 3). In addition, Kaplan-Meier survival analysis of SC/ASC patients revealed that the average survival time was highly correlated with histological differentiation, tumor size, TNM stage, lymph node metastasis, and adjacent tissue invasion (Table 4, *P* < 0.005). Cox multivariate analysis indicated that poor differentiation, tumor size > 3 cm, TNM stage III or IV, lymph node metastases, adjacent tissue invasion, no resection, and CCT2 and PDIA3 positive expression all negatively correlated with post-surgery survival time, and positively correlated with patient mortality rate (Table 5, *P* < 0.05). In addition, CCT2 positivity was found to be an independent prognostic risk factor for SC/ASC (Table 5, *P* = 0.033).

**Table 4:** Relationship between CCT2 and PDIA3 expression and clinicopathological characteristics and average survival of gallbladder SC/ASC patients

**Clinicopathological features**	**Case number**	**Average survival (months)**	**χ**^**2**^	***P***
Gender				
male	19	10.74(6 to 24)	0.767	0.381
female	27	9.85(4 to 24)		
Age (years)				
≤45	3	15.67(8 to 24)	2.023	0.155
>45	43	9.84(4 to 24)		
Pathological types				
SC	26	10.19(4 to 24)	0.223	0.637
ASC	20	10.25(4 to 24)		
Degree of differentiation^a^				
well-differentiated	16	7.04(4 to 11)		
moderately-Differentiated	24	8.92(4 to 18)	19.125	0.000
poorly-differentiated	6	5.83(4 to 9)		
Tumor maximum diameter (cm)				
≤ 3 cm	20	14.35(7 to 24)	31.337	0.620
> 3 cm	26	7.04(4 to 11)		
Gallbladder stones				
no	18	8.22(4 to 12)	3.730	0.053
yes	28	11.50(4 to 24)		
TNM stage				
I + II	12	17.00(9 to 24)		
III	20	9.20(7 to 15)	51.139	0.000
IV	14	5.86(4 to 8)		
Lymph node metastasis				
no	17	14.24(4 to 24)	16.219	0.000
yes	29	7.86(4 to 15)		
Invasion				
no	16	15.75(9 to 24)	32.271	0.000
yes	30	7.27(4 to 12)		
Surgical				
radical		16.64(10 to 24)		
palliative		8.50(6 to 12)	50.165	0.000
biopsy		6.00(4 to 8)		
CCT2				
-	23	12.96(6 to 24)	14.073	0.000
+	33	7.48(4 to 12)		
PDIA3				
-	20	12.75(6 to 24)	7.011	0.008
+	26	8.27(4 to 24)		

**Table 5:** Multivariate Cox regression analysis of survival rate in SC/ASC patients

**Group**	**Factors**	**RC**	**SE**	**Wald**	**RR**	***P***	**95% confidence interval**	
							**Lower limit**	**Upper limit**
Pathologic type	SC/ASC	.028	.379	.005	1.028	.941	.489	2.162
Degree of differentiation	well-/moderately-/poorly-differentiated	.856	.357	5.749	2.354	.016	1.169	4.738
Tumor diameter (cm)	≤ 3 cm/ > 3 cm	2.225	.863	6.647	9.253	.010	1.705	50.223
Gallbladder stones	no/yes	.669	.405	2.729	1.952	.099	.883	4.318
TNM stage	I + II / III / IV	1.231	.457	7.256	3.425	.007	1.398	8.387
Lymph node metastasis	no/yes	1.645	.578	8.100	5.181	.004	1.669	16.085
Adjacent invasion	no/yes	2.498	.796	9.848	12.158	.002	2.554	57.868
Surgical approach	radical/palliative/biopsy	2.762	.822	11.290	15.831	.001	3.161	79.291
CCT2	−/+	1.001	.547	3.349	2.721	.067	.931	7.950
PDIA3	−/+	0.889	.418	4.523	2.433	.033	1.072	5.519

*RC* regression coefficient, *SE* standard error, *Wald* Wald χ^2^, *RR* relative risk.

**Figure 4:**
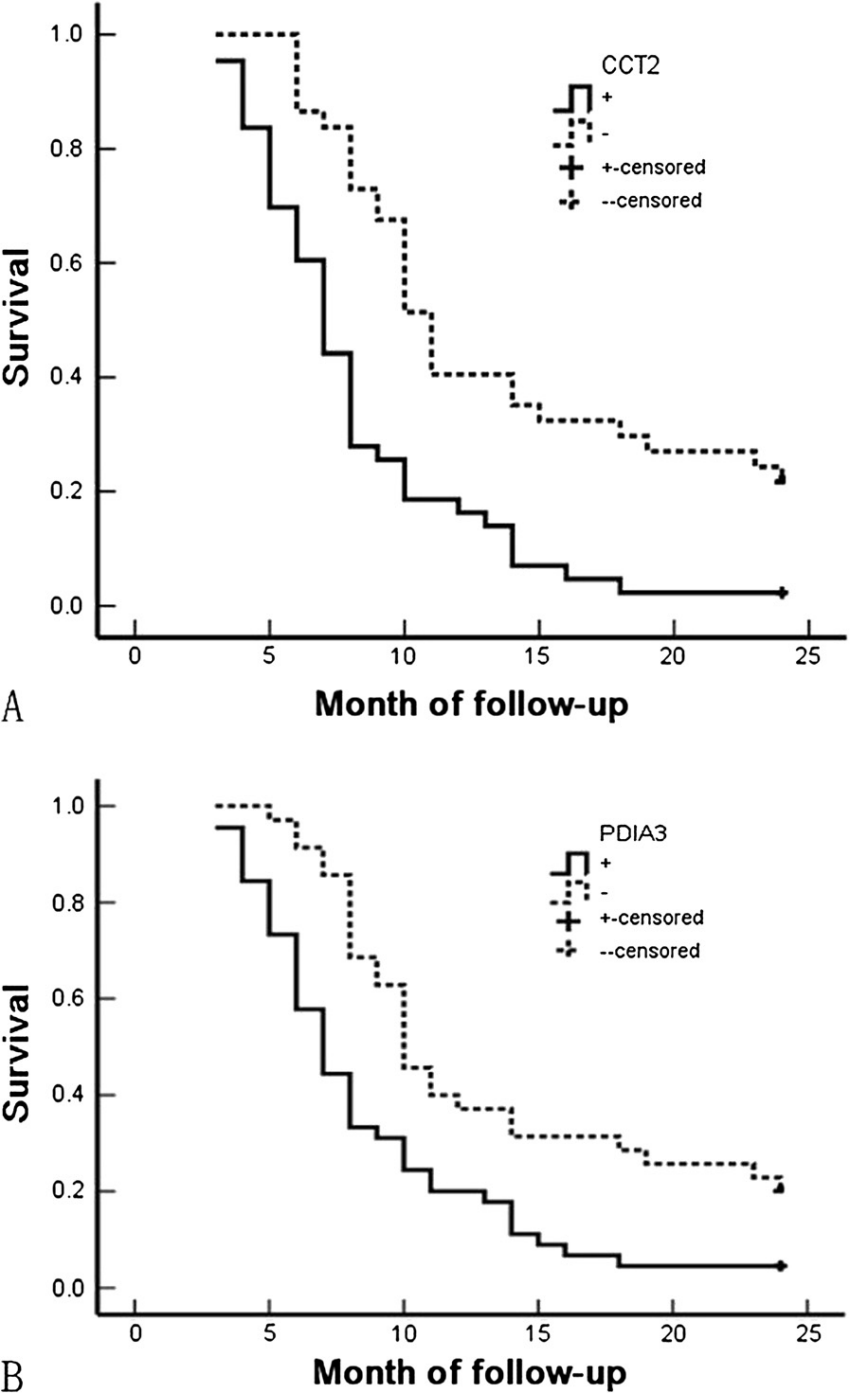
**Correlation between CCT2 or PDIA3 expression and overall survival of patients with gallbladder AC. (A)** Kaplan-Meier plots of overall survival of patients with gallbladder AC according to CCT2 expression in their cancers (*P* < 0.001). **(B)** Kaplan-Meier analysis of overall survival of patients with gallbladder AC according to PDIA3 expression in their cancers (*P* = 0.001).

Similar to the findings in SC/ASC, we also found that the expression of CCT2 and PDIA3 significantly correlated with the shorter survival time of AC patients (*P* < 0.001 and *P* = 0.001 for CCT2 and PDIA3, respectively, in Table 6, and Figure 4). In addition, poor differentiation, tumor size > 3 cm, TNM stage III or IV, lymph node metastases, adjacent tissue invasion, no resection, and positive expression of CCT2 and/or PDIA3 were all negatively correlated with post-surgery survival rate, and positively correlated with patient mortality rate (Table 7, *P* < 0.05). In contrast to that in SC/ASC, both CCT2 expression and PDIA3 expression were independent prognostic risk factors for AC (Table 7, *P* = 0.04 and *P* = 0.014 for CCT2 and PDIA3, respectively).

**Table 6:** Relationship between CCT2 and PDIA3 expression and clinicopathological characteristics and average survival of gallbladder AC patients

**Clinicopathological features**	**Case number**	**Average survival (months)**	**χ**^**2**^	***P***
Gender				
male	26	9.58(3 to 24)	2.567	0.109
female	54	11.30(3 to 24)		
Age (years)				
≤ 45	16	10.81(4 to 24)	0.003	0.956
> 45	64	10.72(3 to 24)		
Degree of differentiation^a^				
well-differentiated	27	15.07(5 to 24)		
moderately-differentiated	25	10.60(4 to 24)	32.501	0.000
poorly-differentiated	28	6.68(3 to 14)		
Tumor maximum diameter (cm)				
≤ 3 cm	50	13.70(6 to 24)	68.283	0.000
> 3 cm	30	5.80(3 to 10)		
Gallbladder stones				
no	42	10.19(3 to 24)	0.246	0.620
yes	38	11.34(4 to 24)		
TNM stage				
I+II	21	18.96(5 to 24)		
III	38	9.29(6 to 15)	105.825	0.000
IV	21	5.14(3 to 7)		
Lymph node metastasis				
no	30	16.27(4 to 24)	42.372	0.000
yes	50	7.42(3 to 14)		
Invasion				
no	31	16.68(7 to 24)	55.535	0.000
yes	49	6.98(3 to 11)		
Surgical				
radical	26	18.31(10 to 24)		
palliative	28	8.64(6 to 11)	113.141	0.000
biopsy	26	5.42(3 to 9)		
CCT2				
-	37	13.78(6 to 24)	17.876	0.000
+	43	8.12(3 to 24)		
PDIA3				
-	35	13.46(5 to 24)	11.604	0.001
+	45	8.62(3 to 24)		

**Table 7:** Multivariate Cox regression analysis of survival rate in AC patients

**Group**	**Factors**	**RC**	**SE**	**Wald**	**RR**	***P***	**95% confidence interval**	
							**Lower limit**	**Upper limit**
Degree of differentiation	Well-/moderately-/poorly- differentiated	1.040	.497	4.379	2.829	.036	1.068	7.494
Tumor diameter (cm)	≤ 3 cm/> 3 cm	1.003	.410	5.985	2.726	.014	1.221	6.090
Gallbladder stones	no/yes	.077	.246	.098	1.080	.754	.667	1.749
TNM stage	I + II / III / IV	1.227	.419	8.576	3.411	.003	1.500	7.754
Lymph node metastasis	no/yes	1.183	.454	6.790	3.264	.009	1.341	7.947
Adjacent invasion	no/yes	1.021	.507	4.055	2.776	.044	1.028	7.499
Surgical approach	radical/palliative/biopsy	1.849	.424	19.017	6.353	.000	2.768	14.586
CCT2	−/+	.853	.296	8.305	2.347	.004	1.314	4.192
PDIA3	−/+	.682	.278	6.018	1.978	.014	1.147	3.411

*RC* regression coefficient, *SE* standard error, *Wald* Wald **χ**^**2**^, *RR* relative risk.

## Discussion

In this study, we analyzed the expression of CCT2 and PDIA3 by IHC staining in 46 SC/ASC samples as well as in 80 AC samples, and found that both CCT2 expression and PDIA3 expression were significantly correlated with tumor progression and shorter survival time for patients with SC/ASC and AC.

For the first time, we found that positive CCT2 expression in both SC/ASC and AC subtypes was significantly associated with clinicopathological features, including large tumor size, high TNM stage, and lymph node metastasis. Moreover, positive expression of CCT2 in AC also correlated with poor differentiation and adjacent tissue invasion. These results, taken together, suggest an important role for CCT2 in GBC progression. Given the critical role of CCT2 in assisting the folding of actin, tubulin, and other cytosolic proteins [14,15], we assumed that the increased CCT2 expression in GBC cells might be critical for these cells to meet the increased levels of protein folding required during fast growth. In line with this hypothesis, overall survival analysis showed that the survival time of patients with positive CCT2 expression was significantly shorter than that of patients with CCT2 negative expression. Moreover, CCT2 is identified as an independent prognostic biomarker for both SC/ASC and AC patients in our multivariate survival analysis. In addition to its up-regulation in GBC, CCT2 up-regulation has been reported in other human tumors and is related to drug-resistance of tumor cells [20]. Therefore, CCT2 in GBC may also contribute to chemotherapeutic resistance, which is important for the clinical treatment of GBC and warrants further study.

Up-regulation of PDIA3 expression is linked to cellular stress response [30]. PDIA3 is also connected to the apoptotic process and has an anti-apoptotic effect in the melanoma cells after induction of endoplasmatic reticulum stress [31]. Overexpression of PDIA3 has been reported in various tumors, including melanomas, cervical carcinoma, ovarian carcinoma, esophageal squamous cell carcinoma, and hepatocellular carcinoma, and is associated with tumor progression, metastasis and poor patient survival rate [22-24,32-35]. Consistent with these studies, we found that positive PDIA3 expression was significantly associated with higher TNM stage, surrounding tissue invasion and lymph node metastasis in both SC/ASC and AC subtypes of GBC. In addition, PDIA3 expression was correlated with poor differentiation and large tumor size in the AC subtype. Furthermore, positive PDIA3 expression was significantly related to the overall survival of patients with both SC/ASC and AC. Therefore, the expression level of PDIA3 in AC and SC/ASC reflected the progression and clinical behavior of gallbladder cancer and might be an important biomarker of poor prognosis. It should be noted that in some cancer types, down-regulation of PDIA3 is related to tumor progression. For example, a lack of PDIA3 expression has been correlated with increased tumor invasion and the advanced stage of gastric cancer, and has therefore been proposed as a good prognostic marker [36]. In addition, PDIA3 expression is down-regulated in metastatic prostate cancer, suggesting that down regulation of PDIA3 might play a role in the late onset of prostate cancer progression [37]. Therefore, the expression of PDIA3 and its role in tumor progression seems to be tumor type specific.

Although there are differences in histopathological features and incidence between SC/ASC and AC subtypes of GBC, our study provided evidence that both SC/ASC and AC have common molecular features, that is, positive expression of CCT2 and PDIA3, and that the expression of CCT2 and PDIA3 in tumor cells correlates with tumor progression and poor prognosis.

## Conclusion

In conclusion, positive expression of CCT2 and PDIA3 are associated with large tumor size, high TMN stage, lymph node metastasis, adjacent tissue invasion, and poor prognosis of SC/ASC and AC. Expression of CCT2 and PDIA3 may reflect the progression and clinical behavior of GBC. CCT2 and PDIA3 could be important diagnostic and prognostic biomarkers for both the SC/ASC and AC subtypes of GBC.

## Abbreviations

GBC: gallbladder cancer; AC: adenocarcinoma; SC/ASC: squamous cell carcinoma/adenosquamous carcinoma; CCT2: chaperonin containing TCP1, subunit 2; PDIA3: protein disulfide isomerase A3; AFP: alpha-fetoprotein; FBS: fetal bovine serum; IHC: immunohistochemistry.

## Competing interests

The authors declare that there are no competing financial interests in this study.

## Authors’ contributions

QZ, YY and ZLY designed the study, performed the sequence alignment, and drafted the manuscript. JHL performed the sequence alignment. LFL designed the study and performed the statistical analysis. GXZ and SLC conceived the study, participated in its design, and coordinated it, and helped to draft the manuscript. All authors have read and approved the final manuscript.

## References

[B1] Hsing AW Gao YT Rashid A Gallstones and the risk of biliary tract cancer: a population-based study in China British J Cancer 2007 11 1577 1582 10.1038/sj.bjc.6604047 PMC2360257 18000509

[B2] Hawkins WG DeMatteo RP Jarnagin WR Jaundice predicts advanced disease and early mortality in patients with gallbladder cancer Ann Surg Oncol 2004 11 310 315 10.1245/ASO.2004.03.011 14993027

[B3] Kim WS Jang KT Choi SH Clinicopathologic analysis of adenosquamous/squamous cell carcinoma of the gallbladder J Surg Oncol 2011 11 239 242 10.1002/jso.21813 21337551

[B4] Chan KM Yu MC Lee WC Adenosquamous/squamous cell carcinoma of gallbladder J Surg Oncol 2007 11 129 134 10.1002/jso.20576 17262729

[B5] Mingoli A Brachini G Petroni R Squamous and adenosquamous cell carcinoma of carcinomas of the gallbladder J Exp Clin Cancer Res 2005 11 143 150 15943044

[B6] Kondo M Dono K Sakon M Adenosquamous carcinoma of the gallbladder Hepatogastroenterology 2002 11 1230 4 12239911

[B7] Oohashi Y Shirai Y Wakai T Adenosquamous carcinoma of the gallbladder warrants resection only if curative resection is feasible Cancer 2002 11 3000 3005 10.1002/cncr.10578 12115390

[B8] Nishihara K Nagai E Izumi Y Adenosquamous carcinoma of the gallbladder: a clinicopathological, immunohistochemical and flow-cytometric study of twenty cases Jpn J Cancer Res 1994 11 389 399 10.1111/j.1349-7006.1994.tb02372.x 7911122 PMC5919463

[B9] Kubota H Hynes G Willison K The chaperonin containing t-complex polypeptide 1 (TCP-1). Multisubunit machinery assisting in protein folding and assembly in the eukaryotic cytosol Eur J Biochem 1995 11 3 16 10.1111/j.1432-1033.1995.tb20527.x 7601114

[B10] Kubota H Hynes G Willison K The eighth Cct gene, Cctq, encoding the theta subunit of the cytosolic chaperonin containing TCP-1 Gene 1995 11 231 236 10.1016/0378-1119(94)00880-2 7890169

[B11] Dekker C Stirling PC McCormack EA The interaction network of the chaperonin CCT EMBO J 2008 11 1827 1839 10.1038/emboj.2008.108 18511909 PMC2486426

[B12] Amit M Weisberg SJ Nadler-Holly M Equivalent mutations in the eight subunits of the chaperonin CCT produce dramatically different cellular and gene expression phenotypes. J Mol Biol 2010 11 532 543 10.1016/j.jmb.2010.06.037 20600117

[B13] Grantham J Brackley KI Willison KR Substantial CCT activity is required for cell cycle progression and cytoskeletal organization in mammalian cells Exp Cell Res 2006 11 2309 2324 10.1016/j.yexcr.2006.03.028 16765944

[B14] Cowan NJ Lewis SA Type II chaperonins, prefoldin and the tubulin-specific chaperones Adv Prot Chem 2001 11 73 104 10.1016/s0065-3233(01)59003-8 11868281

[B15] Frydman J Folding of newly translated proteins in vivo: the role of molecular chaperones Annu Rev Biochem 2001 11 603 647 10.1146/annurev.biochem.70.1.603 11395418

[B16] Yokota S Yamamoto Y Shimizu K Increased expression of cytosolic chaperonin CCT in human hepatocellular and colonic carcinoma Cell Stress Chaperones 2001 11 345 350 10.1379/1466-1268(2001)006<0345:IEOCCC>2.0.CO;2 11795471 PMC434417

[B17] Coghlin C Carpenter B Dundas SR Characterization and over-expression of chaperonin t-complex proteins in colorectal cancer J Pathol 2006 11 351 357 10.1002/path.2056 16981251

[B18] Qian-Lin Z Ting-Feng W Qi-Feng C Inhibition of cytosolic chaperonin CCTζ-1 expression depletes proliferation of colorectal carcinoma in vitro J Surg Oncol 2010 11 419 423 10.1002/jso.21625 20872946

[B19] Malta-Vacas J Nolasco S Monteiro C Translation termination and protein folding pathway genes are not correlated in gastric cancer Clin Chem Lab Med 2009 11 427 431 19284299 10.1515/CCLM.2009.091

[B20] Lin YF Tsai WP Liu HG Intracellular beta-tubulin/chaperonin containing TCP1-beta complex serves as a novel chemotherapeutic target against drug-resistant tumors Cancer Res 2009 11 6879 6888 10.1158/0008-5472.CAN-08-4700 19690144

[B21] Lindquist JA Jensen ON Mann M ER-60, a chaperone with thiol-dependent reductase activity involved in MHC class I assembly EMBO J 1998 11 2186 2195 10.1093/emboj/17.8.2186 9545232 PMC1170563

[B22] Brocke P Garbi N Momburg F HLA-DM, HLA-DO and tapasin: functional similarities and differences Curr Opin Immunol 2002 11 22 29 10.1016/S0952-7915(01)00294-1 11790529

[B23] Chay D Cho H Lim BJ ER-60 (PDIA3) is highly expressed in a newly established serous ovarian cancer cell line, YDOV-139 Int J Oncol 2010 11 399 412 20596667 10.3892/ijo_00000688

[B24] Teramoto R Minagawa H Honda M Protein expression profile characteristic to hepatocellular carcinoma revealed by 2D-DIGE with supervised learning Biochim Biophys Acta 2008 11 764 772 10.1016/j.bbapap.2008.02.011 18359300

[B25] Lwin ZM Yip GW Chew FT Downregulation of ER60 protease inhibits cellular proliferation by inducing G1/S arrest in breast cancer cells *in vitro* Anat Rec (Hoboken) 2012 11 410 416 10.1002/ar.22413 22266712

[B26] Krynetskaia NF Phadke MS Jadhav SH Chromatin-associated proteins HMGB1/2 and PDIA3 trigger cellular response to chemotherapy-induced DNA damage Mol Cancer Ther 2009 11 864 872 10.1158/1535-7163.MCT-08-0695 19372559 PMC2684979

[B27] Sanada Y Yoshida K Ohara M Expression of orotate phosphoribosyltransferase in hepatobiliary ans pancreatic carcinoma Pathol Oncol Res 2007 11 105 113 10.1007/BF02893485 17607371

[B28] Chang HJ Yoo BC Kim SW Significances of PML and P53 protein as molecular prognostic markers of gallbladder carcinomas Pathol Oncol Res 2007 11 326 335 10.1007/BF02940312 18158568

[B29] Nemeth Z Szasz AM Somoracz AT Zonula occludin, and E-cadherin protein expression in biliary tract cancer Pathol Oncol Res 2009 11 533 539 10.1007/s12253-009-9150-4 19184677

[B30] Ni M Lee AS ER chaperones in mammalian development and human diseases FEBS Lett 2007 11 3641 3651 10.1016/j.febslet.2007.04.045 17481612 PMC2040386

[B31] Corazzari M Lovat PE Armstrong JL Targeting homeostatic mechanisms of endoplasmic reticulum stress to increase susceptibility of cancer cells to fenretinide-induced apoptosis: the role of stress proteins ERdj5 and ERp57 Br J Cancer 2007 11 1062 1071 10.1038/sj.bjc.6603672 17353921 PMC2360126

[B32] Linge A Kennedy S O'Flynn D Differential expression of fourteen proteins between uveal melanoma from patients who subsequently developed distant metastases versus those who did not Invest Ophthalmol Vis Sci 2012 11 4634 4643 10.1167/iovs.11-9019 22570344

[B33] Liao CJ Wu TI Huang YH Glucose-regulated protein 58 modulates cell invasiveness and serves as a prognostic marker for cervical cancer Cancer Sci 2011 11 2255 2263 10.1111/j.1349-7006.2011.02102.x 21917082

[B34] Kashyap MK Harsha HC Renuse S SILAC-based quantitative proteomic approach to identify potential biomarkers from the esophageal squamous cell carcinoma secretome Cancer Biol Ther 2010 11 796 810 10.4161/cbt.10.8.12914 20686364 PMC3093916

[B35] Cicchillitti L Di Michele M Urbani A Comparative proteomic analysis of paclitaxel sensitive A2780 epithelial ovarian cancer cell line and its resistant counterpart A2780TC1 by 2D-DIGE: the role of ERp57 J Proteome Res 2009 11 1902 1912 10.1021/pr800856b 19714814

[B36] Leys CM Nomura S LaFleur BJ Expression and prognostic significance of prothymosin-alpha and ERp57 in human gastric cancer Surgery 2007 11 41 50 10.1016/j.surg.2006.05.009 17188166

[B37] Dhanasekaran SM Barrette TR Ghosh D Delineation of prognostic biomarkers in prostate cancer Nature 2001 11 822 826 10.1038/35090585 11518967

